# Patterns of inflammatory responses and parasite tolerance vary with malaria transmission intensity

**DOI:** 10.1186/s12936-017-1796-x

**Published:** 2017-04-11

**Authors:** Temitope W. Ademolue, Yaw Aniweh, Kwadwo A. Kusi, Gordon A. Awandare

**Affiliations:** 1 West African Center for Cell Biology of Infectious Pathogens, Department of Biochemistry, Cell and Molecular Biology, College of Basic and Applied Sciences, University of Ghana, Legon, Accra, Ghana; 2 Department of Immunology, Noguchi Memorial Institute for Medical Research, College of Health Sciences, University of Ghana, Legon, Accra, Ghana

**Keywords:** Cytokines, Immunity, Malaria, Parasite tolerance, Transmission intensity

## Abstract

**Background:**

In individuals living in malaria-endemic regions, parasitaemia thresholds for the onset of clinical symptoms vary with transmission intensity. The mechanisms that mediate this relationship are however, unclear. Since inflammatory responses to parasite infection contribute to the clinical manifestation of malaria, this study investigated inflammatory cytokine responses in children with malaria from areas of different transmission intensities (ranging from low to high).

**Methods:**

Blood samples were obtained from children confirmed with malaria at community hospitals in three areas with differing transmission intensities. Cytokine levels were assessed using the Luminex^®^-based magnetic bead array system, and levels were compared across sites using appropriate statistical tests. The relative contributions of age, gender, parasitaemia and transmission intensity on cytokine levels were investigated using multivariate regression analysis.

**Results:**

Parasite density increased with increasing transmission intensity in children presenting to hospital with symptomatic malaria, indicating that the parasitaemia threshold for clinical malaria increases with increasing transmission intensity. Furthermore, levels of pro-inflammatory cytokines, including tumour necrosis factor alpha (TNF-α), interferon-gamma (IFN-γ), interleukin (IL)-1β, IL-2, IL-6, IL-8, and IL-12, decreased with increasing transmission intensity, and correlated significantly with parasitaemia levels in the low transmission area but not in high transmission areas. Similarly, levels of anti-inflammatory cytokines, including IL-4, IL-7, IL-10 and IL-13, decreased with increasing transmission intensity, with IL-10 showing strong correlation with parasitaemia levels in the low transmission area. Multiple linear regression analyses revealed that transmission intensity was a stronger predictor of cytokine levels than age, gender and parasitaemia.

**Conclusion:**

Taken together, the data demonstrate a strong relationship between the prevailing transmission intensity, parasitaemia levels and the magnitude of inflammatory responses induced during clinical malaria.

**Electronic supplementary material:**

The online version of this article (doi:10.1186/s12936-017-1796-x) contains supplementary material, which is available to authorized users.

## Background

In endemic areas, protection against clinical malaria results from repeated exposure to *Plasmodium falciparum* parasites [1, 2], such that individuals residing in holo-endemic areas can tolerate high levels of parasites without showing clinical symptoms. In low transmission areas however, clinical malaria has been associated with low parasite thresholds [3], suggesting that the threshold parasitaemia for clinical malaria differs in children of similar ages who reside in areas with different transmission intensities [4–6]. These patterns demonstrate that the mechanisms of anti-parasite immunity are distinct from those responsible for anti-disease immunity or parasite tolerance.

Increase in the breadth and magnitude of parasite-specific antibody responses following repeated parasite exposures [7] is expected to control parasitaemia, and reduce the incidence of clinical disease [8]. However, this is not always true in high transmission areas, where children could harbour relatively high parasitaemia but remain asymptomatic [1, 2, 7]. Therefore, while adaptive immune responses may adequately account for anti-parasite immunity, the mechanisms for anti-disease immunity or parasite tolerance remain unclear.

Clues to the mechanisms of parasite tolerance may lie in the role of inflammatory cytokines, which have been shown to correlate with the onset of symptomatic disease during *P. falciparum* infection [9–15]. *Plasmodium falciparum* infection causes paroxysmal fever that is triggered by strong pro-inflammatory responses involving pyrogenic cytokines such as interleukin (IL)-1β and tumour necrosis factor alpha (TNF-α) [16]. Although inflammatory responses, including interferon gamma (IFN-γ), IL-12, IL-1β, IL-2, and TNF-α, play important roles that facilitate parasite clearance [9, 17, 18], circulating high levels of these cytokines have been associated with malaria immunopathology [11, 12, 14, 19–23]. Similarly, high levels of pro-inflammatory cytokines released during malaria infection have been associated with several pathologic processes such as sequestration of infected red blood cells (iRBCs) [24, 25], organ-specific inflammation that results in complications such as cerebral malaria [15, 26, 27], and placental malaria [28]. To prevent these deleterious effects, anti-inflammatory cytokines such as IL-10, IL-4, IL-17, and IL-13 are secreted to balance the effects of pro-inflammatory cytokines [29, 30].

The intensity of transmission has been shown to be a major predictor of clinical manifestations and outcomes of malaria in endemic areas [6, 31]. In holo-endemic areas, disease severity is predominantly related to hyperparasitaemia and severe malarial anaemia [6, 31, 32], whereas in low to medium transmission areas, there is a high rate of cerebral malaria [6, 31, 33, 34]. Given the importance of pro-inflammatory mediators in determining manifestations of malaria, this study investigated the relationship between transmission intensity and inflammatory cytokine responses in children with symptomatic malaria. The roles of these factors in influencing the levels of parasitaemia were also examined. The results provide evidence of a strong relationship between transmission intensity and inflammatory responses during acute malaria infection, and suggest that these factors influence the levels of parasitaemia at clinical presentation.

## Methods

### Study sites

Three outpatient hospitals at locations (Kintampo, Navrongo and Accra) representing distinct malaria transmission intensities in Ghana were selected for this study. Kintampo is holo-endemic for malaria with year-round transmission, and an entomological inoculation rate (EIR) of >250 infective bites/person/year [35]. Navrongo is hyperendemic for malaria with seasonal rainfall and transmission (high transmission from May to November, low transmission from December to April) and EIR of 50–250 infective bites/person/year [36]. Accra is the capital city and has a relatively low transmission intensity (<50 infective bites/person/year) that peaks between June and August annually [37]. Samples were collected from 2011 to 2013 during the peak transmission seasons at the respective study sites.

### Participants and sample collection

Ethical approvals were obtained from the ethics committees of the Ghana Health Service, Navrongo Health Research Centre, Kintampo Health Research Centre and Noguchi Memorial Institute for Medical Research, University of Ghana, Accra, Ghana. Participation was voluntary, and written informed consent was obtained from parents/guardians of the children. Study participants were children aged 2–14 years who were showing signs of clinical malaria, and had been referred for malaria tests by the attending physician. Parasitaemia was detected by malaria rapid diagnostic tests (RDTs) and confirmed by microscopic examination of thick and thin blood smears. Parasite density was estimated by counting the number of parasites per 200 white blood cells as previously described [38, 39]. Haemoglobin levels were quantified using an automated haematology analyzer. Before delivery of anti-malarial and/or any other treatment interventions, 5 mL of venous blood was obtained from each child. Plasma samples were separated from whole blood by centrifugation at 2500 rpm (Eppendorf, model: 5810 R) for 10 min and aliquoted into Eppendorf tubes for storage at −80 °C until further experiments. Sample collection, storage and analysis were done using the same protocols and procedures to ensure uniformity and comparability of data from the different hospitals.

### Cytokine assays

Plasma concentrations of cytokines were measured using the highly sensitive xMAP technology (Luminex Corporation), which allows the simultaneous quantification of several biological analytes in a 96-well format. The MILLIPLEX^®^ MAP 13-Plex Kits from Millipore (Merck Group, magnetic beads) were used because of their higher detection accuracy and reproducibility of results compared to other vendors [40]. These kits were used to quantify eight pro-inflammatory cytokines (TNF-α, IFN-γ, IL-1β, IL-2, IL-8, granulocyte monocyte colony stimulating factor (GM-CSF), IL-6 and IL-12p70) and four anti-inflammatory cytokines (IL-4, IL-7, IL-10, IL-13) in duplicate wells for each plasma sample. These analytes were selected based on relevance and association with malaria. The assays were conducted strictly following the manufacturer’s instructions without any modification. The kits used were from the same lot, and the samples were randomly distributed across plates. Prior to assay, samples were thawed and clarified by centrifugation (2000 rpm for 10 min). There were no readings from the background wells while the quality control and the Standards wells were within the specified range of the kits. Samples with percentage coefficient of variation (%CV) >15% were excluded from further analysis. Cytokine detection limits are found in Additional file 1.

### Statistical analyses

Data analyses and graphs were done using GraphPrism version 6.01 (GraphPad Software, Inc.) and Minitab version 17.1.0.0 (Minitab Inc.). After initial normality tests, patients’ demographics and clinical parameters were compared across the three sites either by Pearson’s Chi square (χ^2^) test (to compare proportions in categorical variables) or One-way ANOVA or Kruskal–Wallis H (K–W) test (for continuous data sets), depending on normality of data. An across-site comparison of cytokine levels was performed with the K–W test, while Dunn’s multiple comparison test was used to reveal between-site pairwise significant differences. Spearman’s correlation analyses were performed for associations of cytokines levels with age and parasite density. In addition, a Spearman’s correlation matrix was built to detect associations between cytokines. Multiple linear regression analyses were conducted to detect the variable(s) that is/are the best predictor(s) of cytokine levels. For the regression models, cytokine levels served as the outcome variables while parasitaemia, age, gender and transmission intensity served as the predictor variables. Statistical significance was generally set at *P* < 0.05, however, after Bonferroni’s procedure, the critical value (α) of the regression models was adjusted to 0.004.

## Results

### Demographic and clinical characteristics of patients across the study sites

To investigate the role of inflammatory responses in the development of malaria parasite tolerance in endemic areas, this study examined the relationship between transmission intensity and the patterns of cytokine production in children with malaria. Using a cross-sectional approach, a total of 173 children who tested positive for malaria by RDTs and microscopy were recruited from three community hospitals in three areas with varying transmission intensities: Accra (*N* = 71) < Navrongo (*N* = 44) < Kintampo (*N* = 58). The proportions of both sexes were comparable across the study sites (*P* = 0.270; Table 1). Children in Accra were relatively older than those in Navrongo (*P* = 0.010) and Kintampo (*P* = 0.025), however, the ages of children from Navrongo and Kintampo did not differ (*P* = 0.999). Parasitaemia reflected the intensity of transmission, with children from Kintampo having higher parasitaemia compared with those in Accra (*P* = 0.005) and Navrongo (*P* = 0.070), although these differences were not statistically significant for Navrongo (Table 1; Additional file 2). Haemoglobin levels decreased as transmission intensity increased (*P* = 0.007), with children from Accra having significantly higher haemoglobin levels compared with those from Kintampo (*P* = 0.008). Although haemoglobin levels in children in Accra were also higher than those in Navrongo, this difference was not statistically significant (*P* = 0.076). Children from Navrongo and Kintampo had comparable haemoglobin levels (*P* = 0.545). The median temperature at clinic also decreased with increasing transmission intensity (*P* < 0.001), with children from Accra having significantly higher median temperature compared to Navrongo (*P* < 0.001) and Kintampo (*P* < 0.001). Children in Navrongo also had higher median temperature than those from Kintampo (*P* < 0.001). Since these children were recruited at presentation to hospital with symptoms of malaria, the pattern of parasitaemia suggests that the parasitaemia threshold for clinical manisfestation of infection seems to increase with increasing transmission intensity.

**Table 1:** Demographic and clinical parameters of patients across the study sites

Characteristics	Accra	Navrongo	Kintampo	*P* value
Participants (number)	71	44	58	–
Female (number, %)	35.3	50.0	43.2	0.270^b^
Median age (IQR), years	6 (4–9)	4 (3–6)	4 (2–6)	0.005^a^*
Median parasitemia (IQR), per µL	21,805 (7,172–64,355)	46,351 (18,524–66,679)	70,215 (11,342–209,335)	0.004^a^*
Mean hemoglobin level (IQR), g/dL	10.5 (9.0–11.6)	9.8 (8.8–10.9)	9.6 (8.1–11.2)	0.007^c^*
Median temperature (IQR), °C	38.8 (38.0–39.4)	38.0 (37.0–39.0)	37.1 (36.5–38.3)	0.0001^a^*

*IQR* interquartile range

*Significant difference

^a^Kruskal–Wallis H test

^b^χ^2^ test

^c^One-way ANOVA

### Levels of pro-inflammatory mediators decline with increasing transmision intensity

**Fig. 1:**
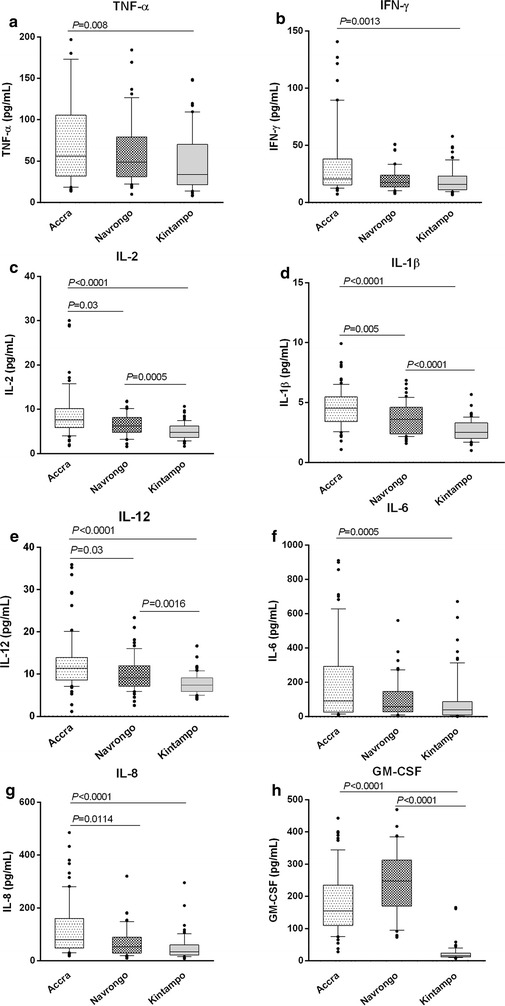
Pattern of pro-inflammatory responses to malaria infection across different transmission sites. Plasma levels of pro-inflammatory cytokines **a** tumour necrosis factor (TNF)-α, **b** interferon (IFN)-γ, **c** interleukin (IL)-2, **d** IL-1β, **e** IL-12, **f** IL-6, **g** IL-8, and **h** granulocyte macrophage colony stimulating factor (GM-CSF), were quantified in children with malaria in three areas of Ghana with varying malaria transmission intensities (Accra < Navrongo < Kintampo). Comparisons across sites were performed using Kruskal-Wallis H test with Dunn’s posthoc test (Accra *N* = 71; Navrongo *N* = 44; Kintampo *N* = 58). Data are presented as box plots where *boxes* represent the inter-quartile ranges, while the whiskers represent the 10th and 90th percentiles. The lines across the *boxes* indicate the median values, while *closed circles* represent outliers

The secretion of pro-inflammatory cytokines during malaria infection has been shown to culminate in the clinical manifestations of disease [13, 14]. Consequently, the role of cytokine levels in mediating parasite tolerance was investigated in children exposed to different malaria transmission intensities. Quantification of levels of pro-inflammatory cytokines, including TNF-α, IFN-γ, IL-1β, IL-2, IL-8, IL-6, IL-12, and GM-CSF in children with malaria in the three transmission areas revealed a pattern of decreasing cytokine levels with increasing transmission intensity (Accra > Navrongo > Kintampo; Fig. 1). Levels of all pro-inflammatory cytokines were significantly lower in children from Kintampo compared to those in Accra (*P* < 0.005 for all cytokines; Fig. 1a–h). In addition, levels of all pro-inflammatory cytokines except IFN-γ (*P* = 0.834), IL-8 (*P* = 0.056) and IL-6 (*P* = 0.260) were lower in the Kintampo group compared to the Navrongo group (*P* < 0.05 for all comparisons; Fig. 1). Given that parasitaemia levels increased with increasing transmission intensity (Table 1), the reverse pattern of pro-inflammatory cytokine suggest that lower cytokine responses appear to favour increased parasite tolerance.

### Levels of anti-inflammatory mediators also decline with increasing transmission intensity

**Fig. 2:**
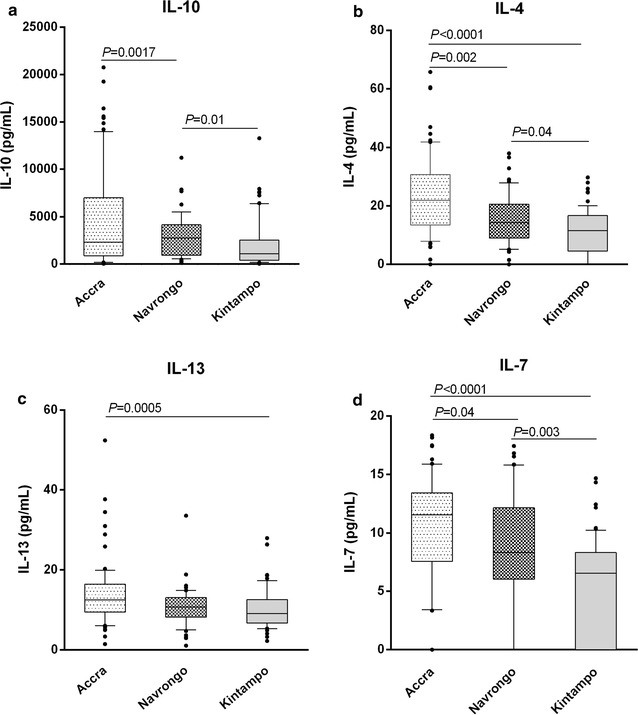
Pattern of anti-inflammatory responses to malaria infection across the different transmission areas. Plasma levels of anti-inflammatory cytokines **a** interleukin (IL)-10, **b** IL-4, **c** IL-13, and **d** IL-7, were quantified in children with malaria in three areas of Ghana with varying malaria transmission intensities (Accra < Navrongo < Kintampo). Comparisons across sites were performed using Kruskal-Wallis H test with Dunn’s posthoc test (Accra *N* = 71; Navrongo *N* = 44; Kintampo *N* = 58). Samples below the detection limits were assigned a concentration of zero, including 12 samples for IL4 (Accra = 3, Navrongo = 1, Kintampo = 8), and 33 samples for IL7 (Accra = 6, Navrongo = 7, Kintampo = 20). Data are presented as box plots where *boxes* represent the inter-quartile ranges, while the whiskers represent the 10th and 90th percentiles. The *lines* across the boxes indicate the median values, while *closed circles* represent outliers

Since pro-inflammatory responses are usually followed by the secretion of anti-inflammatory mediators to balance their effects [13, 14, 29, 30], the levels of four key anti-inflammatory cytokine including IL-4, IL-7, IL-10, and IL-13 were also examined. The pattern observed was similar to that of the pro-inflammatory cytokines, with cytokine levels decreasing with increasing transmission intensity across the three areas (Accra > Navrongo > Kintampo; Fig. 2). Specifically, levels of all four cytokines were significantly lower in children recruited in Kintampo compared to those in Accra (*P* < 0.01 for all comparisons; Fig. 2). Taken together, these data buttress the patterns observed for pro-inflammatory responses, and further confirm an association between transmission intensity and the magnitude of inflammatory responses induced during clinical malaria, and suggest a likely association with parasitaemia levels.

### Correlation between parasitaemia and pro-infammatory cytokine levels varies with transmission intensites

**Fig. 3:**
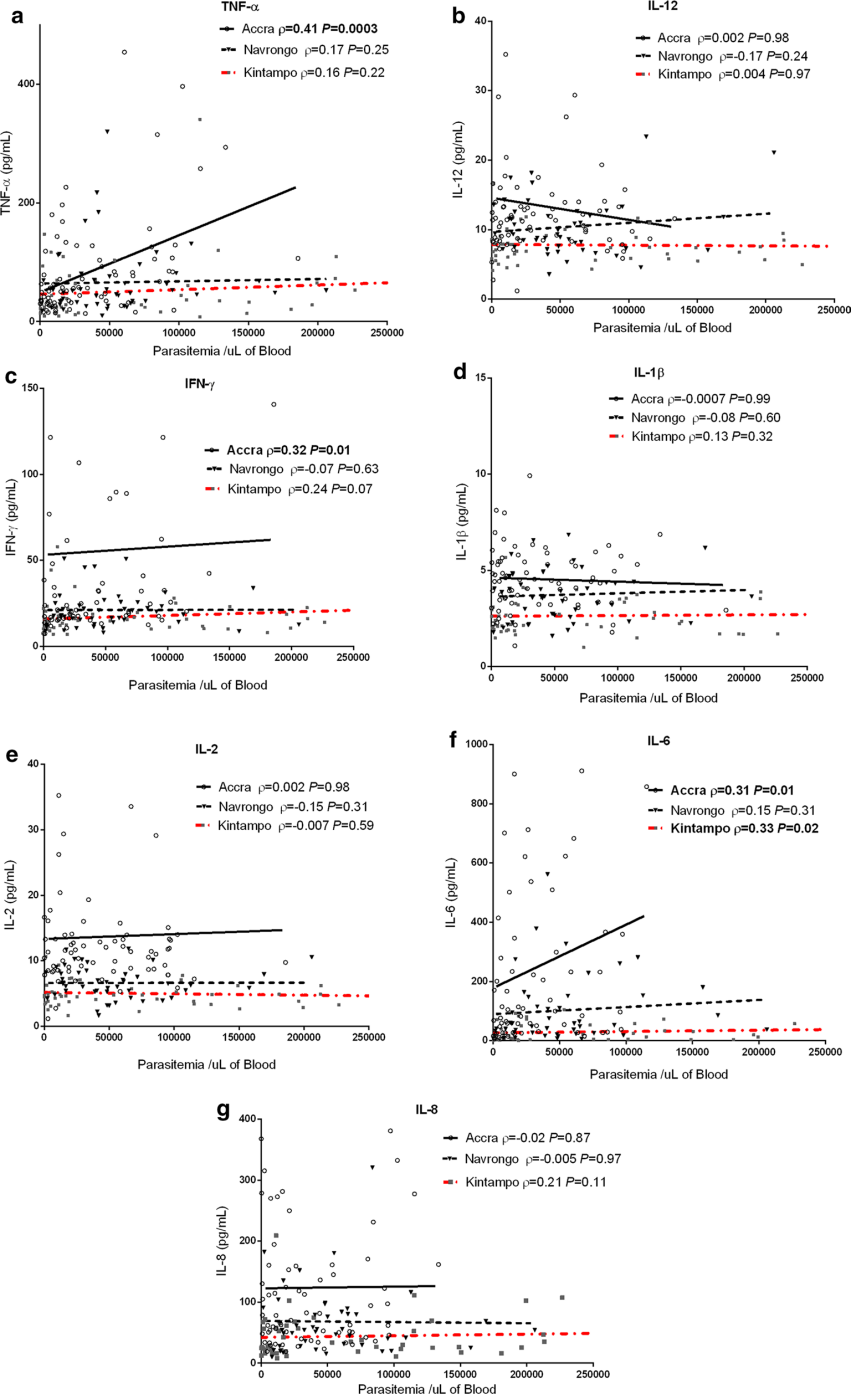
Association between pro-inflammatory cytokines and parasite density across the sites. The relationships between parasite density in children with malaria and plasma levels of pro-inflammatory cytokines **a** tumour necrosis factor (TNF)-α **b** interleukin (IL)-12, **c** interferon (IFN)-γ, **d** IL-1β, **e** IL-2, **f** IL-6 and **g** IL-8, were examined using Spearman’s rank correlation test. *P* values in bold type indicate statistical significance. Samples below the detection limits were excluded from the analysis. (ρ = Spearman’s correlation coefficient)

Given the strong relationship between cytokine levels and transmission intensity, the correlations between levels of pro-inflammatory mediators and parasitaemia were directly examined in each of the three sites. There were significant positive correlations between parasite density in children with malaria and levels of key pro-inflammatory cytokines, including TNF-α, IFN-γ and IL-6 (Fig. 3). However, these correlations were observed in the Accra group only, with none showing significant correlation in the Navrongo group, and only IL-6 showing a significant correlation in the Kintampo group (Fig. 3). Since malaria parasite antigens induce the production of pro-inflammatory cytokines [14, 16, 41, 42], these results are further evidence of increased parasite tolerance in the higher transmission areas, where further increases in parasite levels above a certain threshold no longer augment cytokine production.

### Limited correlations between parasitemia and anti-inflammatory cytokine levels

**Fig. 4:**
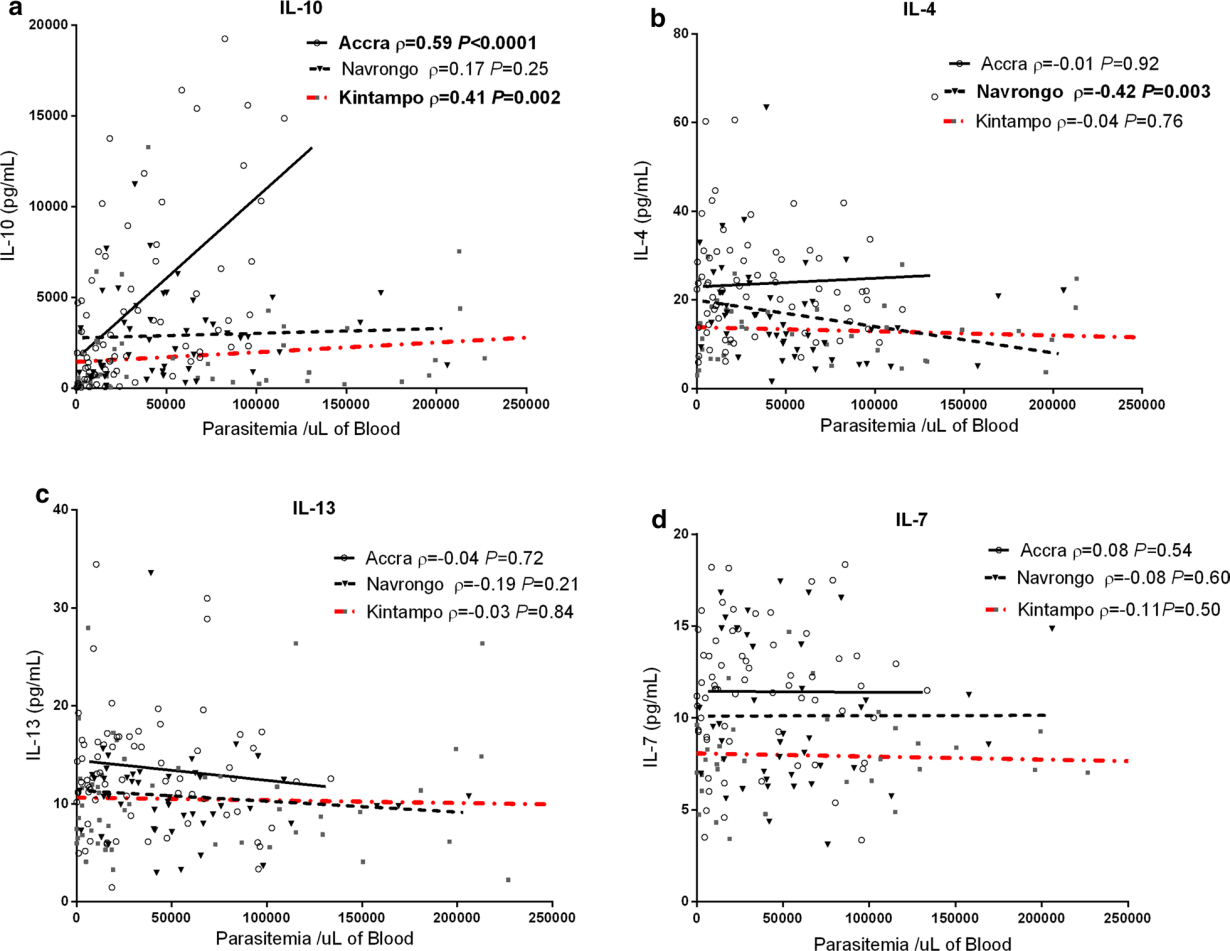
Association between anti-inflammatory cytokines and parasite density across the sites. The relationships between parasite density in children with malaria and plasma levels of anti-inflammatory cytokines **a** interleukin (IL)-10, **b** IL-4, **c** IL-13, and **d** IL-7, were examined using Spearman’s rank correlation test. *P* values in bold type indicate statistical significance. Samples below the detection limits were excluded from the analysis. (ρ = Spearman’s correlation coefficient)

Subsequently, the correlations between anti-inflammatory cytokines and parasite burden were examined in children with malaria across the different transmission areas. Unlike the patterns observed with the pro-inflammatory cytokines, the anti-inflammatory cytokines did not generally show significant correlation with parasitaemia in any of the endemic areas (Fig. 4). However, IL-10, which is considered a critical anti-inflammatory mediator in regulating the pro-inflammatory response during malaria [29, 43, 44], showed significant correlation with parasite density in children residing in Accra and Kintampo (Fig. 4). This correlation was particularly strong in the Accra group, which is consistent with the correlation observed for the key pro-inflammatory cytokines. In addition, IL-4 showed a significant association with parasitaemia in children from Navrongo, however, this correlation was negative (Fig. 4).

### Associations between parasite density and cytokine levels are independent of age across sites

Cytokine levels during parasitic infections, including malaria, have been shown to vary with age [45]. Since age significantly differed between sites (Table 1), the study subsequently determined whether the age difference affected cytokine responses during acute malaria infection. The results showed limited associations between age and cytokine levels in this cohort, with IFN-γ correlating negatively with age in Navrongo and Kintampo (Additional file 3), while IL-8 and IL-4 showed positive correlations with age in Navrongo and Accra, respectively (Additional file 3). Thus, contrary to previous reports where age was found to significantly affect levels of cytokines during malaria infection [6, 13, 45, 46], age did not seem to be a major determinant of plasma levels of cytokines across the sites.

### Transmission intensity is the major predictor of cytokine responses

**Fig. 5:**
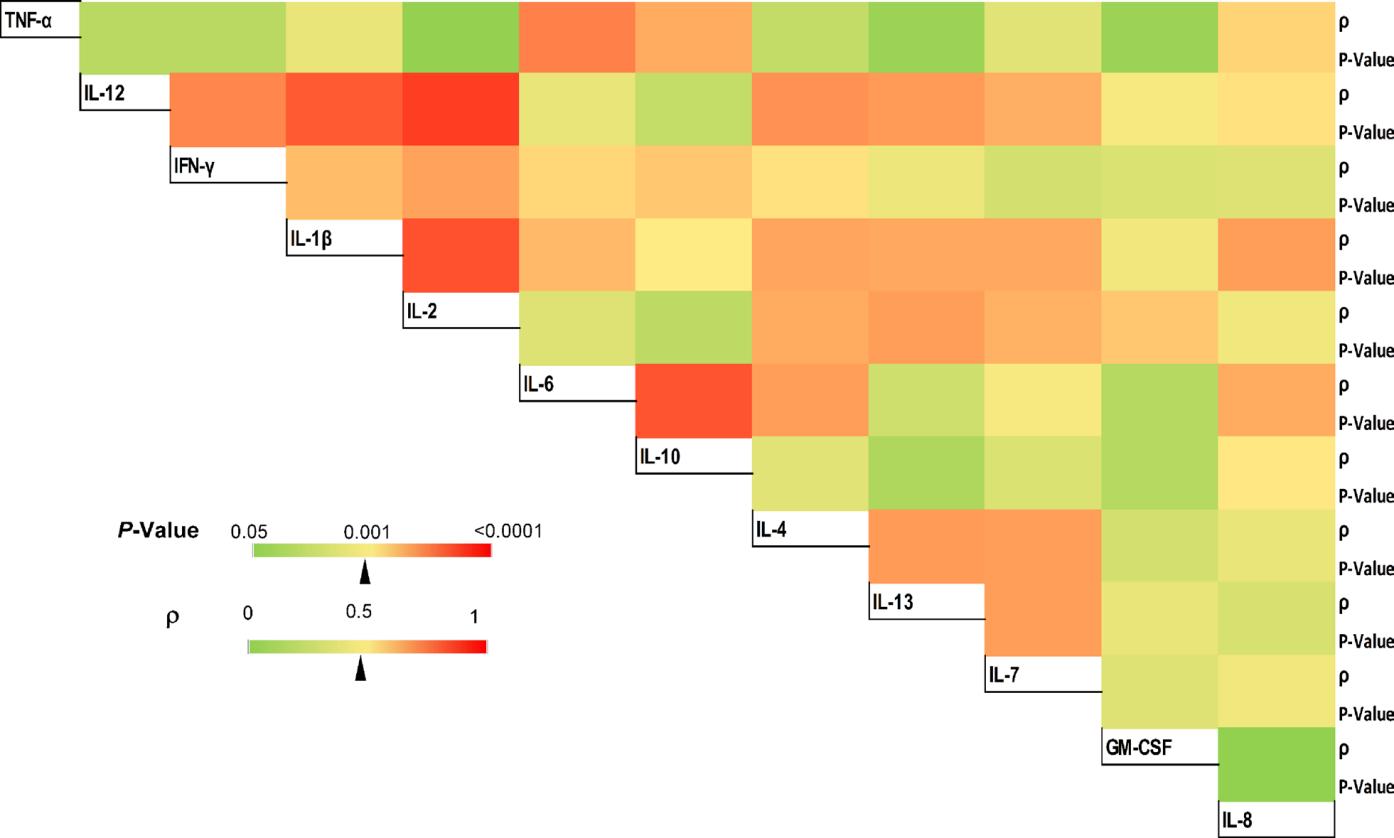
Correlation matrix showing the relationships between cytokines across the sites. The interrelationships between the levels of inflammatory cytokines in children with malaria were examined by Spearman’s correlation test, and the results are summarized in a colour matrix. The strength of correlation between pairs of cytokines are illustrated on a colour scale, where the least statistically significant relationships are coloured green while the most significant are in *red*. (ρ = Spearman’s correlation coefficient)

Using multiple linear regression analyses, the relative contributions of age, gender, parasite density, and transmission intensity as predictors of cytokine levels was examined. These analyses revealed that study site (coded in order of increasing transmission intensity) was the strongest predictor of all cytokine levels, except GM-CSF, for which sex was the best predictor (see F values in Table 2). Transmission intensity had negative regression coefficients in all the regression models (negative β-weights, Additional file 4); indicating an inverse relationship with cytokine levels. In addition, the interrelationships among the cytokines across the sites were investigated, and this showed that cytokines generally correlated positively with each other (Fig. 5). Most significantly, the key pro-inflammatory cytokines, including IFN-γ, IL-12 and IL-2 strongly correlated with each other, and a strong association was also found between TNF-α and IL-6 (Fig. 5).

**Table 2:** Summary of multiple linear regression analysis for predictors of cytokine levels

	TNF-α		IL-12		IFN-γ		IL-1β		IL-2		IL-6		IL-8		IL-4		IL-10		IL-13		IL-7		GM-CSF	
Variables (df, error)	F	*P*	F	*P*	F	*P*	F	*P*	F	*P*	F	*P*	F	*P*	F	*P*	F	*P*	F	*P*	F	*P*	F	*P*
Age (1,171)	0.48	0.490	2.02	0.15	0.37	0.54	1.41	0.23	1.74	0.18	1.41	0.236	0.76	0.38	2.00	0.159	0.00	0.954	2.98	0.086	0.23	0.635	2.35	0.127
Parasitemia (1,171)	0.80	0.37	0.44	0.510	0.25	0.61	0.02	0.88	1.47	0.22	0.02	0.880	0.29	0.590	0.29	0.59	*8.99*	*0.003* ^#^*	1.79	0.183	0.24	0.625	1.27	0.261
Sex (1,171)	0.82	0.366	0.39	0.53	0.01	0.914	0.10	0.750	3.18	0.076	0.10	0.750	0.01	0.937	0.08	0.782	0.04	0.837	0.18	0.67	0.12	0.729	*6.64*	0.011^*#*^
Transmission Intensity (2,171)	2.43	0.091	6.51	0.002^#^*	6.38	0.001^#^*	7.36	0.001^#^*	9.26	<0.0001^#^*	7.36	0.001^#^*	9.78	<0.0001^#^*	8.09	<0.0001^#^*	13.26	<0.0001^#^*	5.04	0.007^*#*^	10.02	<0.0001^#^*	1.74	0.178

Table summarizing the multiple linear regression analyses. Cytokine levels were the outcome variables, while age, parasitemia, sex and transmission intensity (sites) were the predictor variables. Transmission intensity was the major significant predictor of cytokine levels

F = F-statistic

df = Degree of freedom

Error = Residual degrees of freedom

^#^Statistically significant predictor

*Significant after Bonferroni’s *P* value adjustment

## Discussion

Previous studies have established that individuals exposed to endemic malaria transmission can harbour high parasitaemia without clinical symptoms [6, 31, 47], suggesting that the threshold parasitaemia for symptomatic malaria in high transmission areas is higher than that in low-to-medium transmission areas [1, 2, 10, 39]. Data presented here support this phenomenon, whereby increasing transmission intensity was associated with increasing parasite densities in children presenting to hospital with symptomatic malaria. Therefore, it was hypothesized that the regulation of pro-inflammatory responses is a mechanism that accounts for the differences in parasite tolerance in individuals exposed to different transmission intensities. This hypothesis is based on established knowledge that pro-inflammatory responses during infection are characterized by the release of a cascade of soluble immune mediators including cytokines and chemokines that cause fever, and other signs of malaria [48]. The results show that pro-inflammatory responses decreased with increasing transmission intensity (Accra > Navrongo > Kintampo). Consistent with the decreasing levels of pyrogenic cytokines, axillary temperature in the children with malaria decreased with increasing transmission intensity, indicating a decreasing intensity of fever.

Interestingly, significant correlations between parasite density and cytokine levels were observed among children with malaria in Accra only, suggesting that this relationship seems to disappear in higher transmission areas. This assertion was supported by the multiple linear regression analyses, which revealed that transmission intensity was the strongest predictor of cytokine responses during acute malaria infection. These findings suggest that higher parasitaemia thresholds for symptomatic malaria in areas of intense malaria transmission may be explained by controlled pro-inflammatory responses, and milder fevers, which consequently delay clinical symptoms until higher parasite densities are attained. On the contrary, lower thresholds of parasitaemia in low transmission areas could be due to a more aggressive pro-inflammatory response against low parasitaemia, leading to more severe fevers and faster onset of clinical manifestation.

High parasitaemia would mean high levels of parasite associated antigens such as glycophosphatidylinositol (GPI) anchors [42, 49, 50], and high levels of damage associated molecular patterns (DAMPs) such as haem from red blood cells [51], which consequently, should induce corresponding high levels of pro-inflammatory response, but such corresponding stimulation was not observed in the high transmission sites. Therefore, tolerance of comparatively higher parasitaemia in areas of intense malaria transmission may be as a result of refractoriness to stimulation from prolonged continuous exposure to parasites and parasite antigens [5, 52]. Previous studies have demonstrated that prolonged stimulation of CD4^+^ T-cells with high level of antigens mediate adaptive peripheral tolerance, which is characterized by unresponsiveness to further stimulation, with an evident decrease in the secretion of TNF-α, IFN-γ, IL-2, and IL-6 [5, 52–54]. A parallel observation has been described in sepsis, where it was demonstrated that at certain level of stimulation in vitro, cells become refractory to stimulation with bacterial endotoxin, showing no further secretion of pro-inflammatory cytokines [55–57].

In high transmission areas, more frequent infections would mean an almost ‘chronic’ state of infection [2]. Under this condition, peripheral CD4^+^ T-cells are exhausted [58] from persistent stimulation with high levels of parasite associated antigens. In addition, the loss of a Vγ9^+^δ2^+^ T cell subset, which rapidly expands and become activated during *P. falciparum* infection, was recently shown to be associated with repeated infections [59]. This sub-set of T-cells has been shown to secrete high levels of TNF-α and IFN-γ upon stimulation with iRBCs [60]. Perhaps, low levels of TNF-α and IFN-γ observed in the high transmission sites is due to the reduction of this T-cell subset. On the other hand, lower exposure in low transmission areas means that each infection is a separate acute event, which culminates in responses similar to those observed in naïve individuals (i.e., heightened pro-inflammatory response). This result is further buttressed by a recent report which independently demonstrated that pro-inflammatory responses during acute malaria infection increases with decreasing exposure; being highest in naïve adults, followed by immigrants with extended loss of *P. falciparum* exposure, and being lowest in semi-immune individuals residing in an endemic area [61].

Alternatively, there appears to be a mechanism that dampens pro-inflammatory responses [62] in children that have been repeatedly exposed to the parasite [63] through suppression of IL-12 production. Low levels of IL-12 in the high transmission areas could be a result from suppression by ingested haemozoin [64], due to the reported high levels of haemozoin-containing monocytes [65] in children residing in holo-endemic areas. In addition, evidence of suppression of T-cell cytokine responses was recently demonstrated in murine models of malaria [63], where a distinct sub-set of IL-27-secreting Foxp3^−^CD11a^+^CD49d^+^ malaria antigen-specific CD4^+^ T-cells inhibit the production of IL-2, which consequently may dampen IL-12 secretion, resulting in clonal depletion of Th1 cells [63]. Similarly, the development of humoral immune responses appear to be associated with better control of pro-inflammatory responses in children with malaria from Malawi [42].

## Conclusion

Altogether, findings from this study represent significant new knowledge about the mechanisms of malaria pathogenesis and parasite tolerance. The data also provide evidence and understanding of malaria parasite tolerance, an issue of utmost importance in the context of malaria control and eradication since the adverse effects of malaria resurgences are not known. While these findings need to be confirmed by additional investigations of the cellular responses underlying the patterns of cytokine production, data presented here have implications for characterizing the pathophysiology of *P. falciparum* amidst decreasing transmission intensity.

## Additional files

**Additional file 1.** Assay Sensitivities (minimum detectable concentrations, pg/ml) of analytes.

**Additional file 2.** The distribution of age and clinical parameters of patients’ across the study sites. (A) and (C) Red line across indicates mean while error bars represent standard deviation (One-way ANOVA, with Tukey’s posthoc multiple comparison test to reveal pairwise significant differences) (B) Data is presented as a box plot with whiskers and outliers. The box represents the inter-quartile range, while the whiskers represent the 10th and 90th percentiles. The line across the box indicates the median value, closed circles represent outliers (Kruskal–Wallis test, with Dunn’s posthoc multiple comparison test to reveal pairwise significant differences).

**Additional file 3.** Association between cytokines and age across the sites.

**Additional file 4.** The regression coefficients of transmission intensity as a predictor of cytokine levels.
