# Supplementary material for: Patterns of inflammatory responses and parasite tolerance vary with malaria transmission intensity
Source: Malar J. 2017 Apr 11;16:145. doi: 10.1186/s12936-017-1796-x (PMC5387356; doi:10.1186/s12936-017-1796-x)
Supplement: Supplementary file 1 — Additional file 1. Assay Sensitivities (minimum detectable concentrations, pg/ml) of analytes. [file 12936_2017_1796_MOESM1_ESM.docx]

**Supplementary data**

**Additional file 1**

**Additional file 1: Assay Sensitivities (minimum detectable concentrations, pg/mL) of analytes.**

| **Cytokine** | **MinDC (pg/ml)** | **MinDC+2SD (pg/ml)** |
| --- | --- | --- |
| GM-CSF | 7.5 | 15.0 |
| IFNγ | 0.8 | 1.1 |
| IL-10 | 1.1 | 1.6 |
| IL-12P70 | 0.6 | 1.0 |
| IL-13 | 1.3 | 1.9 |
| IL-1β | 0.8 | 1.0 |
| IL-2 | 1.0 | 1.6 |
| IL-4 | 4.5 | 7.1 |
| IL-6 | 0.9 | 1.3 |
| IL-7 | 1.4 | 2.4 |
| IL-8 | 0.4 | 0.7 |
| TNFα | 0.7 | 1.1 |
