# Supplementary material for: Patterns of inflammatory responses and parasite tolerance vary with malaria transmission intensity
Source: Malar J. 2017 Apr 11;16:145. doi: 10.1186/s12936-017-1796-x (PMC5387356; doi:10.1186/s12936-017-1796-x)
Supplement: Supplementary file 2 — Additional file 2. The distribution of age and clinical parameters of patients’ across the study sites. (A) and (C) Red line across indicates mean while error bars represent standard deviation (One-way ANOVA, with Tukey’s posthoc multiple comparison test to reveal pairwise significant differences) (B) Data is presented as a box plot with whiskers and outliers. The box represents the inter-quartile range, while the whiskers represent the 10th and 90th percentiles. The line across the box indicates the median value, closed circles represent outliers (Kruskal–Wallis test, with Dunn’s posthoc multiple comparison test to reveal pairwise significant differences). [file 12936_2017_1796_MOESM2_ESM.docx]

**Supplementary data**

**Additional file 2**

**

**

**Additional file 2:** **The distribution of age and clinical parameters of patients’ across the study sites.** (A) and (C) Red line across indicates mean while error bars represent standard deviation (One-way ANOVA, with Tukey’s posthoc multiple comparison test to reveal pairwise significant differences) (B) Data is presented as a box plot with whiskers and outliers. The box represents the inter-quartile range, while the whiskers represent the 10th and 90th percentiles. The line across the box indicates the median value, closed circles represent outliers (Kruskal-Wallis test, with Dunn’s posthoc multiple comparison test to reveal pairwise significant differences).
