# Supplementary material for: Patterns of inflammatory responses and parasite tolerance vary with malaria transmission intensity
Source: Malar J. 2017 Apr 11;16:145. doi: 10.1186/s12936-017-1796-x (PMC5387356; doi:10.1186/s12936-017-1796-x)
Supplement: Supplementary file 3 — Additional file 3. Association between cytokines and age across the sites. [file 12936_2017_1796_MOESM3_ESM.docx]

**Supplementary data**

**ADDITIONAL FILE 3**

**Additional file 3: Association between cytokines and age across the sites.**

|  | Accra | | Navrongo | | Kintampo | |
| --- | --- | --- | --- | --- | --- | --- |
|  | **ρ** | ***P*-value** | **ρ** | ***P*-value** | **ρ** | ***P*-value** |
| TNF-α | 0.05 | 0.68 | -0.1808 | 0.2189 | -0.2346 | 0.0737 |
| IL-12 | 0.05745 | 0.6317 | -0.1513 | 0.3099 | -0.08965 | 0.4995 |
| IFN-γ | -0.09447 | 0.4299 | **-0.3603** | **0.0129** | **-0.3065** | **0.0182** |
| IL-1β | 0.0129 | 0.5827 | 0.08829 | 0.5551 | 0.07812 | 0.5565 |
| IL-2 | 0.08760 | 0.4644 | -0.08347 | 0.5770 | 0.06827 | 0.6074 |
| IL-6 | 0.07311 | 0.5446 | -0.05439 | 0.7165 | -0.2088 | 0.1158 |
| IL-8 | 0.05386 | 0.6532 | **0.3039** | **0.0378** | -0.2229 | 0.0898 |
| IL-10 | 0.056 | 0.6399 | -0.09598 | 0.5210 | -0.1046 | 0.4304 |
| IL-4 | **0.25** | **0.0385** | -0.2055 | 0.1612 | -0.08534 | 0.5516 |
| IL-13 | -0.082 | 0.489 | -0.182 | 0.2202 | -1719 | 0.1930 |
| IL-7 | 0.174 | 0.1607 | -0.2782 | 0.074 | -0.1997 | 0.2107 |

**ρ =Spearman’s correlation coefficient.**
