# Supplementary material for: Patterns of inflammatory responses and parasite tolerance vary with malaria transmission intensity
Source: Malar J. 2017 Apr 11;16:145. doi: 10.1186/s12936-017-1796-x (PMC5387356; doi:10.1186/s12936-017-1796-x)
Supplement: Supplementary file 4 — Additional file 4. The regression coefficients of transmission intensity as a predictor of cytokine levels. [file 12936_2017_1796_MOESM4_ESM.docx]

**Supplementary data**

**ADDITIONAL FILE 4**

| Cytokine | Site | Regression coefficient, β | *P* |
| --- | --- | --- | --- |
| TNF-α | Navrongo | -23.7 | 0.14 |
|  | Kintampo | -32.3 | **0.03** |
| IL-12 | Navrongo | -6.94 | **0.011** |
|  | Kintampo | -8.93 | **0.001** |
| IFN-γ | Navrongo | -293.1 | **0.003** |
|  | Kintampo | -278.7 | **0.003** |
| IL-1β | Navrongo | -2.59 | **0.011** |
|  | Kintampo | -3.574 | **< 0.0001** |
| IL-2 | Navrongo | -4.67 | **0.002** |
|  | Kintampo | -5.63 | **< 0.0001** |
| IL-6 | Navrongo | -218.3 | **0.007** |
|  | Kintampo | -317.1 | **< 0.0001** |
| IL-8 | Navrongo | -90.2 | **0.002** |
|  | Kintampo | -112.9 | **<0.0001** |

| Cytokine | Site | Regression coefficient, β_o_ | *P* |
| --- | --- | --- | --- |
| IL-4 | Navrongo | -6.69 | **0.016** |
|  | Kintampo | -10.91 | **<0.0001** |
| IL-10 | Navrongo | -2281 | **0.004** |
|  | Kintampo | -3862 | **<0.0001** |
| IL-13 | Navrongo | -3.36 | **0.007** |
|  | Kintampo | -3.16 | **0.009** |
| IL-7 | Navrongo | -1.349 | 0.065 |
|  | Kintampo | -3.487 | **<0.0001** |
| GM-CSF | Navrongo | 232 | 0.064 |
|  | Kintampo | 102 | 0.398 |

**Additional file 4: Regression coefficients of transmission intensity as a predictor of cytokine levels**

β_o_ = Regression coefficient for study sites, using Accra as reference.

Average R^2^ for all regression models = 10.4%

***Bold type indicates*** statistical significant
